# Cadaveric and Ultrasound-Guided Evaluation of Two Needling Approaches Targeting the Pectoralis Minor Muscle: A Pilot Feasibility Study

**DOI:** 10.3390/jfmk11010121

**Published:** 2026-03-16

**Authors:** José L. Sánchez-Sánchez, Pedro Belón-Pérez, Xavier Grevol-Coll, Miguel Robles-García, Gustavo Plaza-Manzano, César Fernández-de-las-Peñas, Laura Calderón-Díez

**Affiliations:** 1 Department of Physical Therapy, Universidad de Salamanca, 37007 Salamanca, Spain; jlsanchez@usal.es; 2 Institute of Biomedical Research of Salamanca (IBSAL), 37007 Salamanca, Spain; lauca@usal.es; 3 Real Madrid C.F., 28036 Madrid, Spain; pebelon@gmail.com; 4 Department of Physical Therapy, Clinic on the Go. Menorca, 07760 Ciutadella, Illes Balears, Spain; xaviergrevol@hotmail.com; 5 Department of Anatomy and Histology, Universidad de Salamanca, 37007 Salamanca, Spain; mroblesgarcia@usal.es; 6 Radiology, Rehabilitation and Physiotherapy Department, Universidad Complutense de Madrid, 28040 Madrid, Spain; gusplaza@ucm.es; 7 Instituto de Investigación Sanitaria del Hospital Clínico San Carlos, 28040 Madrid, Spain; 8 Department of Physical Therapy, Occupational Therapy, Physical Medicine and Rehabilitation, Universidad Rey Juan Carlos (URJC), 28922 Madrid, Spain

**Keywords:** pectoralis minor, cadaver, nerve, pleura, risk, dry needling

## Abstract

**Background**: The pectoralis minor muscle can be a source of musculoskeletal-related chest pain by contributing to thoracic outlet syndrome. Needling interventions applied to chest wall muscles have an inherent risk of puncturing sensitive structures, e.g., the pleura. **Objective**: The objective of this study was to preliminarily investigate the safety and accuracy of two needling approaches targeting the pectoralis minor muscle. **Methods**: A pincer- and flat-needle approach targeting the pectoralis minor muscle was conducted in five Thiel-embalmed cadavers and 10 healthy volunteers by an experienced and a novice clinician. The needle was inserted until the clinician considered that the pectoralis minor muscle was reached. Each clinician conducted 10 needle insertions with each approach. In cadavers, the accuracy of needle placement was identified with both ultrasound imaging and anatomical dissection. In healthy volunteers, needle placement accuracy was evaluated with ultrasound imaging. **Results**: Accurate needle penetration of the pectoralis minor muscle was 80–90% and 40–70% for experienced and novice clinicians, respectively, with the pincer approach. One pleural puncture was observed in one cadaver specimen with this approach by the novice clinician. Accurate needle penetration of the pectoralis minor muscle was 100% and 90% for experienced and novice clinicians, respectively, with the flat approach. The novice clinician required 3.5 times longer to perform the flat approach than the experienced clinician. **Conclusions**: The results of this pilot feasibility study suggest that a pincer-needle approach seems to be less accurate than the flat-needle approach and substantially more error-prone for a novice clinician, which, in a clinical context, could pose a potential risk of pneumothorax based on the pleural puncture observed in one cadaver specimen. In contrast, our preliminary results revealed that the flat-needle approach could have better accuracy and safety, particularly when performed under real ultrasound guidance.

## 1. Introduction

Approximately half of chest pain consultations attended in primary care emergency services have a musculoskeletal origin [1]. The prevalence of chest pain of musculoskeletal origin has been estimated to range from 10% to 30% [2]. In fact, muscular problems have been described as one of the most frequent causes of musculoskeletal-related chest pain in clinical practice [3]. The anterior part of the chest wall is anatomically covered by the pectoralis major and pectoralis minor muscles. The pectoralis minor is a deep, thin, flat muscle that originates at the costal cartilage margins of the third to fifth ribs and inserts into the superomedial border of the coracoid process [4]. The pectoralis major is a large, fan-shaped muscle with three heads (clavicular, sternal and abdominal) originating at the sternal half of the clavicle, the sternum, the manubrium, the costal cartilage of the upper six ribs, and the external oblique aponeurosis and inserting into the lateral lip of the intertubercular groove of the humerus bone and the glenohumeral joint capsule [4]. The anatomic relationship between the origin of the pectoralis major and minor muscles is highly variable, making both muscles interconnected [5].

The pectoralis minor, described in the literature as a “pseudo-anginal” muscle, is the one most frequently involved in thoracic outlet syndrome, giving rise to the so-called pectoralis minor syndrome [6]. Pectoralis minor syndrome is considered as a subtype of thoracic outlet syndrome where the brachial plexus and/or its vascular component are compressed as they pass under this muscle at the chest, causing pain, tingling, numbness, muscle weakness, or swelling in the upper extremity. This syndrome is traditionally associated with repetitive upper-limb activities, which can lead to shortening (potential fibrosis) of the pectoralis minor muscle, a reduction in the volume of the retro-pectoralis minor space, and subsequent brachial plexus compression causing neurogenic syndrome or vascular compression causing vascular symptoms [7].

Among the etiologies of musculoskeletal chest pain, myofascial pain syndrome was described several decades ago, but it has been an underestimated and overlooked source of chest pain [8]. Myofascial pain is featured by the presence of myofascial trigger points (TrPs), “i.e., hyperirritable spots within a taut band of skeletal muscle that are painful on compression, stretch, overload, or contraction of the tissue which usually responds with a referred pain that is perceived distant from the spot” [9]. The somatic presentation of pectoralis minor TrPs consists of referred pain spreading to the anterior thorax and shoulder, occasionally following the trajectory of the ulnar nerve [9]. The pain referral from pectoralis minor TrPs can mimic cardiac angina and thus may be considered a differential diagnosis for musculoskeletal chest pain [10].

Musculoskeletal chest pain has traditionally been a difficult area to evaluate and treat. Conservative management is effective in most patients. Injections with local anesthetics or corticosteroids are proposed as a treatment method for musculoskeletal chest pain of joint origin (e.g., costochondral joints), whereas botulinum toxin injections have been proposed for musculoskeletal chest pain of muscular origin (e.g., pectoralis minor, scalene) [11]. In the last decades, there has been increasing interest in the application of needling interventions within the physical therapy scope, e.g., dry needling, percutaneous electrolysis, and percutaneous electrical nerve stimulation (PENS) [12]. These interventions have different musculoskeletal targets such as the muscle, tendon and nerve, respectively [12]. Different case reports using dry needling interventions targeting pectoralis minor TrPs for managing chest pain symptoms have been published [13,14]. Thus, TrP injections are also used for managing noncardiac chest pain related to myofascial TrPs [15].

All these interventions use solid needles during treatment applications. Although different authors describe dry needling as a safe procedure, needle approaches applied to the thorax or chest wall are potentially dangerous due to the risk of pleural puncture. Although most adverse events documented with the application of dry needling are minor [16], iatrogenic pneumothorax is the most frequent major adverse event described in the former literature on dry needling [17,18,19].

In fact, different recommendations proposing the safest position of the patient, an appropriate education of the therapist and the use of anatomical landmarks have been published to guide clinicians and to decrease the risk of puncturing sensitive structures, e.g., pleura or nerve [20,21]. However, Mitchell et al. found that position, body constitution and sex of the patient change tissue depth significantly and should be considered when dry needling the thorax region due to the risk of puncturing the pleura [22].

Ultrasound imaging has been proposed as a diagnostic and therapeutic aid to improve the accuracy and precision of needling interventions. In fact, in the last decade, ultrasound-guided needling procedures have been actively applied for the management of chronic pain conditions [23]. Further, different studies have used ultrasound imaging to determine prediction models based on skin-to-rib distance and anthropometric features of individuals to ensure the safety of dry needling approaches applied to the thoracic region [24,25].

The aim of this pilot feasibility study was to preliminarily determine the potential safety and accuracy of two needling approaches targeting the pectoralis minor muscle on Thiel-embalmed cadavers (anatomical landmarks) and in healthy subjects (under ultrasound guidance).

## 2. Methods

### 2.1. Study Design

This pilot study included a cadaveric and clinical phase where feasibility, accuracy, and the influence of operator experience on procedure reliability were preliminarily investigated. The cadaveric study was first conducted to evaluate the safety and precision of both approaches targeting the pectoralis minor muscle, whereas the clinical phase study was conducted to confirm dry needling targeting the pectoralis minor muscle under ultrasound (US) guidance in a sample of healthy volunteers.

### 2.2. Anatomical Study on Thiel-Embalmed Cadavers

Five cadavers preserved using the Thiel embalming technique were provided by the institutional anatomy laboratory of the Autonomous University of Madrid (Spain). All cadaver specimens were checked to rule out prior thoracic surgery or structural anomalies that could alter the anatomical study.

**Figure 1:**
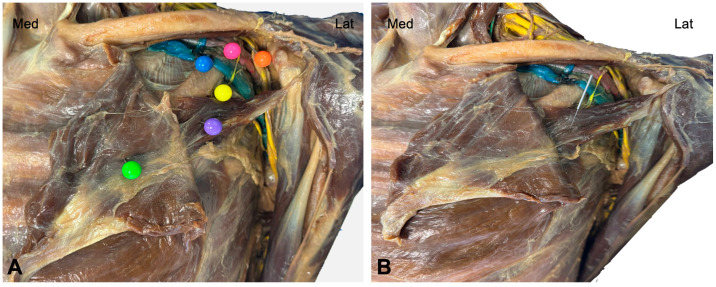
Cadaveric dissection of the thorax and infraclavicular area showing the pectoralis major and minor muscles. (**A**) Pectoralis major muscle (green), pectoralis minor muscle (violet), medial pectoralis nerve (yellow), axillary vein (blue), axillary artery (pink) and brachial plexus (orange). (**B**) Cadaveric dissection showing the accuracy of the needle targeting the pectoralis minor muscle. Med: medial; Lat: lateral.

The skin and subcutaneous fascia of the thorax, anterior shoulder, and neck were excised for visualization of the pectoralis major muscle. The brachial plexus was also exposed at both supraclavicular and infraclavicular levels. The nerve tissue, arteries, and brachial veins had been previously stained to facilitate their identification and visualization. The insertional tendon of the pectoralis major muscle and the clavipectoral fascia were removed from the humerus, allowing access to the pectoralis minor muscle, its insertion on the coracoid process, and its relationship to the neurovascular bundle (Figure 1A).

**Figure 2:**
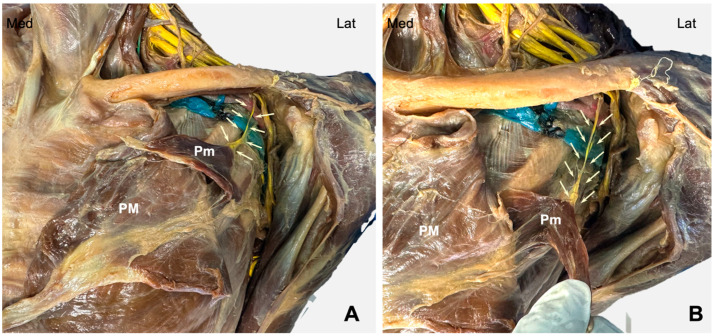
Cadaveric dissection of the thorax and infraclavicular area (**A**) showing the medial pectoralis nerve ((**B**), arrows). PM: pectoralis major muscle; Pm: pectoralis minor muscle; Med: medial; Lat: lateral.

Once the needle was inserted (Figure 1B), the medial pectoral nerve (Figure 2A), running deep to the pectoralis minor muscle and perforating it to reach the pectoralis major muscle, was exposed (Figure 2B).

### 2.3. Participant Enrollment

Ten healthy volunteers (50% women; mean age 21 ± 3 years old) participated in the clinical ultrasound-guided intervention study. Participants were recruited through an announcement at the university. Since this was a validation study, the inclusion criteria required that participants report no upper extremity pain, no symptoms suggestive of neurovascular involvement, and no history of cervical or upper extremity surgery. All procedures were conducted in accordance with the Declaration of Helsinki and were approved by the Human Research Ethics Committee (CBE) of the University of Salamanca, Spain (CBE-1166). All volunteers provided written informed consent before their participation.

### 2.4. Needling Procedures

All procedures on the cadavers were performed strictly using palpation-based anatomical references. Solid filiform needles (40 × 0.3 mm or 50 × 0.3 mm; AguPunt, Barcelona, Spain) were selected according to the estimated tissue thickness of the cadaver. Both pincer- and flat-needling approaches were conducted in all cadavers and healthy participants.

**Figure 3:**
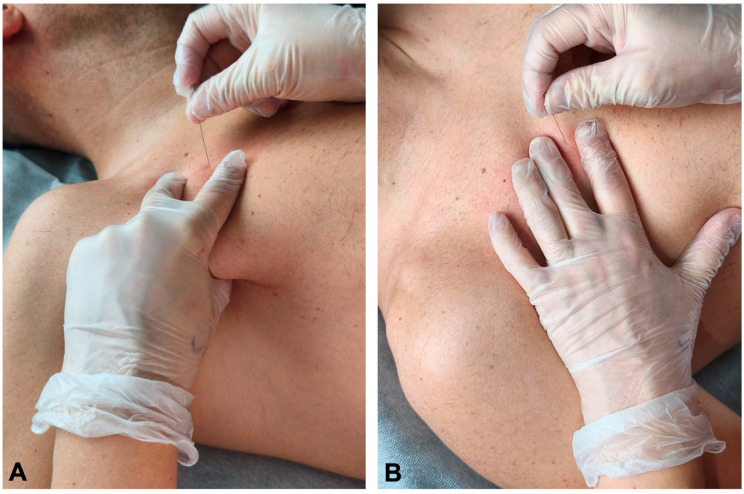
Pincer-palpation needle approach (**A**) and flat-palpation needle approach (**B**) over the pectoralis minor muscle.

For the pincer-needle intervention, the clinician performed a pinch grip using the second and third fingers over the ribcage, superficial to the pectoralis major, while the thumb attempted to contact the deep surface of the pectoralis minor through the anterior axillary wall close to the lateral aspect of the ribs. The thumb was used as a protective reference and targeted point of the tip of the needle. The dominant hand inserted the needle obliquely at approximately 45° to the ribcage, in a craniocaudal and lateromedial direction, toward the thumb (Figure 3A).

The flat needle-intervention targeted the mid-portion of the third rib as follows. The clinician palpated and located the third rib. Once the rib was located, the index and middle fingers were placed over and under the third rib (at the intercostal spaces on either side of the third rib). The needle was then inserted through the pectoralis major at approximately 45° in a craniomedial direction until the superior cortical surface of the third rib was contacted with the tip of the needle (Figure 3B).

### 2.5. Ultrasound Imaging

**Figure 4:**
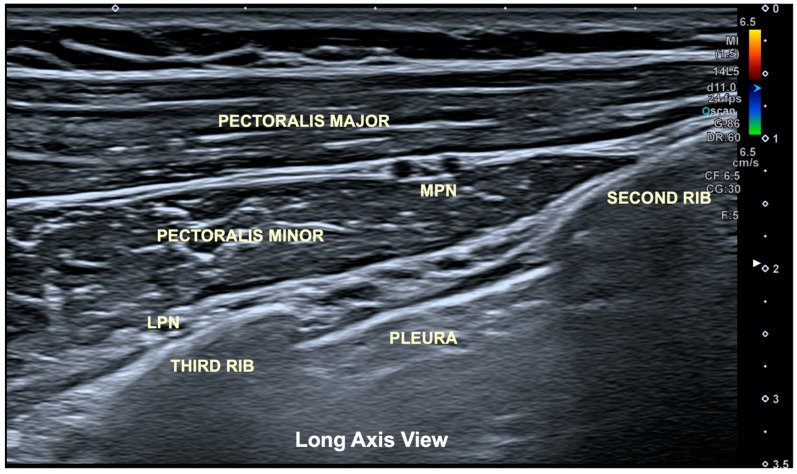
Longitudinal axis ultrasound image showing the “bat sign” (between the second and third rib). Using this anatomical ultrasound view as a reference, the muscle bellies of both pectoralis major and pectoralis minor muscles and the parietal pleura can be properly visualized. MPN: medial pectoral nerve; LPN: lateral pectoral nerve.

An ultrasound (Aplio-A, Canon Medical Systems; Madrid, Spain) with a linear transducer (6–15 MHz) was used to visualize the pectoralis major and pectoralis minor muscles, as well as their fascial boundaries, costal border of the second and third ribs, intercostal muscles and parietal pleura. The clinician looked for the image described as the “bat sign”, which can be observed on a long-axis view. The “bat sign”, popularized by Dr. Lichtenstein, describes the normal appearance of the lung surface in a long-axis ultrasound view: the ribs resemble the wings of a bat (the upper rib as the left wing and the lower rib as the right wing), while the pleural line mimics the body of the bat [26,27] (Figure 4).

The pincer-needle approach (as previously described) was attempted under ultrasound guidance. However, the simultaneous execution of the pincer palpation and manipulation of the probe with the same hand, while inserting the needle with the dominant hand, was not possible. Due to this situation, compromised image quality, safety and accuracy of the needling procedure, the pincer-needle approach in both cadaveric and clinical studies was conducted first by using anatomical landmarks, with a posterior ultrasound verification after insertion.

**Figure 5:**
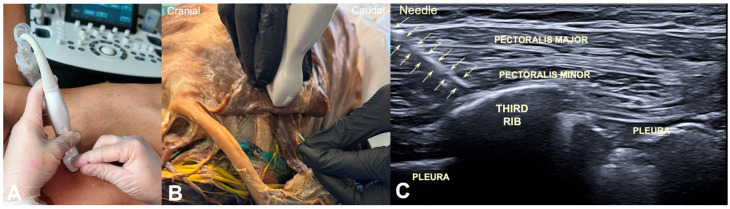
(**A**) Ultrasound-guided in-plane (long-axis) flat-needle approach in a human subject. (**B**) Ultrasound-guided in-plane (long-axis) flat-needle approach in the cadaver showing the pectoralis minor for confirmation. (**C**) Longitudinal-axis ultrasound image showing the needle (arrows) in the muscle belly of the pectoralis minor muscle with the tip towards the third rib. PM: pectoralis major muscle; Pm: pectoralis minor muscle.

In the clinical study, the flat-needle approach was simultaneously ultrasound-guided. The probe was placed longitudinally on the chest wall (Figure 5A). The flat-needle approach (as previously described) in the cadaveric study was conducted first by using anatomical landmarks, with posterior ultrasound verification after insertion (Figure 5B). The needle was introduced at approximately 45° using an in-plane approach, directed craniomedially toward the third rib with a continuous visualization of the tip. The endpoint of insertion was controlled by cortical contact with the superior surface of the third rib, confirming safe depth while ensuring that the needle accurately targeted the pectoralis minor muscle belly without approaching the pleura or neurovascular structures (Figure 5C).

### 2.6. Clinicians

Two clinicians performed both needle approaches. The first one was an experienced therapist with ten years of clinical practice in needling procedures, whereas the second was a novice therapist with one year of clinical experience in needling procedures, including the chest-wall approach. Both clinicians had completed a 100 h post-graduate certification program that included practical training in chest-wall musculature. In addition, for this specific study, both were required to study a manual of standard operating procedures and participate in a 2 h training session.

For the cadaveric study, the clinicians conducted needle insertion based on palpatory anatomical landmarks as described above. Both clinicians were permitted to make as many needle adjustments as needed until they perceived the needle to be located at the pectoralis minor muscle, particularly during the flat-needle approach. A five-minute rest period was allowed between each trial to minimize clinician fatigue. Hence, each clinician performed 20 needle placement attempts, 10 per each approach (pincer and flat palpation). When the clinician considered that the pectoralis minor muscle had been properly reached, with the needle maintained in the targeted area determined by the clinician, an examiner, blinded to the clinician performing the intervention, conducted ultrasound imaging examination to determine if the tip of the needle properly reached the pectoralis minor muscle without puncturing adjacent sensitive thoracic structures.

For the clinical study, due to the impossibility of conducting the pincer-needle approach with appropriate simultaneous ultrasound visualization of the needle in a real individual, this approach was performed as in the cadaveric study, that is, the pincer-needle approach was first done based on anatomical landmarks with posterior ultrasound visualization. Each clinician performed 10 needle placement attempts with a five-minute rest period between insertions. The flat-needle approach was ultrasound-guided and performed in real time as previously described. Each therapist performed one ultrasound-guided attempt per subject, resulting in a total of 10 needle placement attempts per clinician with a five-minute rest period. Again, the clinicians were permitted to make as many adjustments as needed until they perceived the needle position to be accurate.

We recorded the following variables: 1, accurate placement of the needle tip on the pectoralis minor muscle; 2, error in accurate needle placement due to either insufficient or excessive depth; and 3, time required for definitive needle placement (in seconds) during the flat approach.

## 3. Results

### 3.1. Cadaveric Study

Five adult cadavers (three males, mean age: 59 ± 3 years; two females, mean age: 55 ± 5 years) preserved using the Thiel embalming technique were used. During the pincer-needle approach, the experienced clinician reached the pectoralis minor muscle belly accurately in nine out of the 10 insertions.

**Figure 6:**
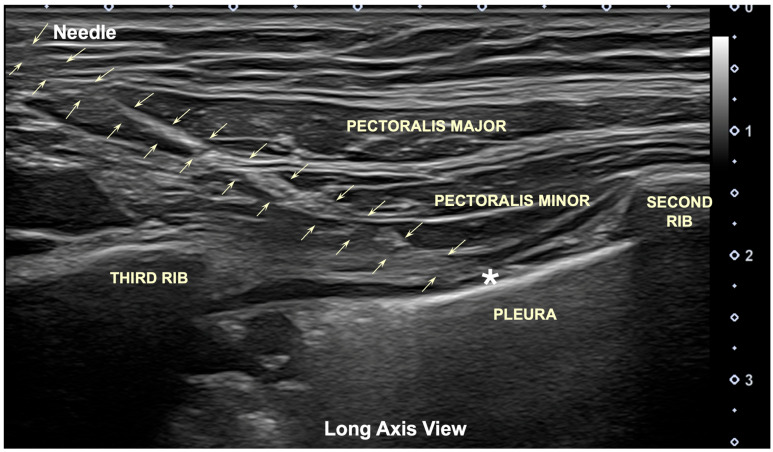
Longitudinal ultrasound image (on a cadaver) of the pincer-needle approach (clamp-on imaging) showing how the needle (arrows) reaches the parietal pleural wall. The needle tip (asterisk) is observed reaching and puncturing the pleura at the intercostal space between the second and third ribs.

In one case, the needle remained insufficiently deep and was placed within the pectoralis major muscle (Table 1) as confirmed by the ultrasound imaging. The novice clinician demonstrated greater variability, with three out of 10 (30%) of the needle insertions being inaccurate (Table 1). Two insertions failed to accurately reach the pectoralis minor muscle because the needle remained in the pectoralis major muscle, while the third insertion exceeded the intended depth, entering the intercostal space and contacting the parietal pleura (Figure 6)—an event that potentially could have provoked a pneumothorax if applied on a living subject.

For the flat-needle approach directed toward the third rib, the experienced clinician accurately placed the tip of the needle on the pectoralis minor muscle during all 10 insertions (Table 2). The novice clinician performed nine out of 10 punctures with accurate placement in the pectoralis minor muscle. In the missing puncture, the needle remained superficial to the targeted muscle, i.e., in the pectoralis major muscle. In this approach, no excessively deep punctures or pleural contact were observed (Table 2). Thus, no vascular or neural injuries resulting from insertions were identified with ultrasound visualization.

### 3.2. Clinical Study

Ten healthy volunteers (50% women; mean age 21 ± 3 years old) were included in the clinical study. For the pincer-needle approach (performed by anatomical landmarks) and posteriorly verified with ultrasound imaging, the experienced clinician accurately reached the pectoralis minor muscle in eight out of 10 insertions, with two inaccurate insertions remaining superficially in the pectoralis major muscle. The novice clinician demonstrated a 60% error rate, with all insufficient-depth insertions remaining superficially in the pectoralis major muscle (Table 1).

For the flat-needle approach toward the third rib, ultrasound-guided insertions were successfully completed in 100% of the cases by both clinicians. All insertions accurately reached the pectoralis minor muscle, confirmed by simultaneous ultrasound visualization. Controlled contact with the third rib served as an effective anatomical safety limit. No pleural contact, vascular involvement, or intercostal penetration occurred. No adverse events were reported, and participant tolerance was excellent. The novice clinician required 3.5 times longer to complete each insertion than the experienced clinician (Table 2).

## 4. Discussion

Dry needling has been shown to be effective for managing different musculoskeletal pain conditions [28]. However, needling interventions targeting the chest wall muscles, particularly the pectoralis minor, require accurate needle placement and should be considered a maneuver of risk due to the proximity of critical structures, particularly the parietal pleura and the lung. In such a scenario, cadaveric studies allow an initial evaluation of the feasibility, accuracy and potential safety of therapeutic interventions with a potential risk of damaging sensitive structures. We hence conducted a pilot feasibility study to preliminarily evaluate the potential accuracy and safety (lack of pleural contact) of two needling approaches targeting the pectoralis minor muscle. By combining cadaveric analysis with an in vivo validation phase, this pilot study provides a preliminary understanding of the anatomical risks associated with pectoralis minor muscle needling, as well as an initial influence of the operator experience. The results suggest that dry needling of the pectoralis minor muscle can be conducted with a pincer or flat-needle approach by experienced clinicians with a preliminary and potential accuracy of up to 90%. On the contrary, the novice operator showed substantially greater variability, including inaccurate depth of the needle and, in one cadaver, even pleural contact.

Several authors proposed a pincer-needle approach for managing pectoralis minor TrPs based just on palpatory anatomical landmarks, as conducted in the current study, and expressly warned of pleura and vascular risk in the anterior thoracic/chest wall area [20,29]. These authors highlighted the need for an oblique-angle needle insertion trajectory for the pincer-needle palpation approach to avoid pleural puncture. Nevertheless, reports of iatrogenic pneumothorax following dry needling in the chest wall/thoracic spine reinforce the necessity for precise control and careful needle angulation, as even minor deviations may compromise pleural integrity [17,18,19]. Although no published pneumothorax cases specifically attributed to pectoralis minor muscle needling have been published, the anatomical configuration of this muscle—near proximity to the parietal pleura—makes the procedure inherently risky when performed without ultrasound imaging guidance. This study confirmed the vulnerability of this anatomical area by showing that a novice clinician, even when instructed and following palpation approaches described in the literature and commonly used in clinical practice [9], may incur dangerous trajectories that exceed the muscular plane and invade the intercostal space.

A key finding of the current study was that the most common errors performed by both clinicians in the pincer-needle approach consisted of the needle remaining superficially within the pectoralis major without properly reaching the pectoralis minor muscle. This occurred in cadaver specimens with significant adipose tissue accumulation in the chest wall. This finding would suggest that increased soft tissue thickness can hinder tactile/manual discrimination of muscular planes with a pincer-palpation approach based just on anatomical landmarks. The presence of subcutaneous tissue alters the relationship between superficial/deep structures; thus, increasing the skin-to-target muscle distance, and leading to error in estimating the required needle insertion depth. These patients would require longer needles, which would increase the potential risk of the procedure. This result reinforces the idea that individual anthropometric variability is a critical factor limiting the reliability of palpation-based needling approaches [20,22], suggesting the relevance of ultrasound guidance to ensure the accuracy and safety of needling approaches in people with greater tissue thickness. Interestingly, this error of placing the needle superficially within the pectoralis major reached up to 60% for the novice clinician when performed on living subjects. The clinical context and the use of “best-case” low-risk cohort individuals, i.e., young asymptomatic people, with less adipose tissue accumulation than the cadaver specimens, could lead to a more prudent approach for avoiding the risk of puncturing the pleura.

The second approach, the flat-needle intervention, demonstrated substantially greater reliability, which was an unexpected finding. In cadavers, the rib served as a stable anatomical barrier limiting excessive depth and reducing pleural risk. Both clinicians accurately reached the pectoralis minor in nearly all insertions, in a safe manner with no pleural penetration. These findings would suggest that the flat approach could be considered intrinsically when performed without ultrasound guidance, reinforcing the desirability of prioritizing trajectories that rely on firm structural landmarks such as the rib. In the clinical study, the presence of a bony landmark and the use of real-time ultrasound guidance eliminated uncertainty regarding needle trajectory and depth, resulting in 100% accuracy for experienced/novice clinicians. Although the novice clinician required more time, this did not affect procedural comfort and did not introduce technical errors. Real-time needle visualization provided a safety window between the tip, the rib and the pleura. Taken together, these findings support the notion that the ultrasound-guided flat-needle approach seems to be safer and more reproducible than the anatomical landmark pincer-needle approach, especially in clinical contexts where pleuropulmonary integrity is mandatory. These results support ultrasound guidance as a key safety measure for pectoralis minor needling, particularly in deep or high-risk thoracic regions. The data also emphasizes the significant role of clinician experience. The novice clinician’s errors in both insufficient and excessive depth underscore the need for supervised learning and training before performing needling interventions in clinical populations [16].

Finally, some limitations of this study should be considered when interpreting the current findings. First, the use of cadaver specimens, whose tissue tone and biomechanical properties differ from those of living subjects, should be considered. In addition, the clinical part of this study represents a “best-case” low-risk cohort, which cannot represent common populations attending clinical practice, particularly older people with higher body mass index. Second, we included a small number of individuals and specimens. No sex differences in needle placement were found. In fact, sex differences in anthropometric data could influence the distance and depth of the muscle. Women typically exhibit higher body fat percentage and a subcutaneous “pear-shaped” distribution in some body areas, e.g., hips, while men display more visceral “apple-shaped” fat around abdominal organs [30]. Third, the number of clinicians was also limited; accordingly, current results should be considered as preliminary. Future studies should explore inter- and intra-rater reliability across broader levels of clinical experience and further refine safe technical parameters for pectoralis minor muscle needling interventions. Finally, this was a pilot feasibility study; accordingly, clinical applicability and effectiveness of both needling approaches should also be evaluated in future studies.

## 5. Conclusions

This pilot feasibility study suggests that needling of the pectoralis minor muscle requires high anatomical precision and should be considered a procedure with risk due to the proximity of the parietal pleura. Preliminary results revealed that a pincer-needle approach showed less accuracy than the flat-needle approach and proved substantially more error-prone for a novice clinician, including instances of insufficient/excessive depth that, in a clinical context, could represent a potential risk of pneumothorax due to pleural puncture in our cadaver, as well as published clinical case reports. In contrast, in this small pilot sample, the flat-needle approach, when performed under real-time ultrasound guidance, appeared safer and more accurate than a palpation-based pincer approach, particularly for a less-experienced clinician. Overall, these preliminary results reinforce the need for individualized risk–benefit assessment of needling interventions targeting the chest wall, accounting for patient morphology, clinician expertise, and the advantage of ultrasound guidance, to ensure procedural safety when considering a needling procedure targeting the pectoralis minor muscle. The results of this pilot feasibility study should be confirmed in large clinical studies.

## Figures and Tables

**Table 1:** Results of the pincer-needle approach.

	Experienced Clinician	Novice Clinician
**Cadaveric Study—Punctures performed (n)**	10	10
Accurate placement on the muscle	9/10 (90%)	7/10 (70%)
Error due to insufficient depth	1/10 (10%)	2/10 (20%)
Error due to excessive depth	0/10 (0%)	1/10 (10%)
Pleural injury	0	1
**Clinical Study—Punctures performed (n)**	10	10
Accurate placement on the muscle	8/10 (80%)	4/10 (40%)
Error due to insufficient depth	2/10 (20%)	6/10 (50%)
Error due to excessive depth	0/10 (0%)	0/10 (0%)
Pleural injury	0	0

**Table 2:** Results of the flat-needle approach.

	Experienced Clinician	Novice Clinician
**Cadaveric Study—Punctures performed (n)**	10	10
Accurate placement on the muscle	10/10 (100%)	9/10 (90%)
Error due to insufficient depth	0/10 (0%)	1/10 (10%)
Error due to excessive depth	0/10 (0%)	0/10 (0%)
Pleural injury	0	0
**Clinical phase—Punctures performed (n)**	10	10
Accurate placement on the muscle	10/10 (100%)	10/10 (100%)
Error due to insufficient depth	0/10 (0%)	0/10 (0%)
Error due to excessive depth	0/10 (0%)	0/10 (0%)
Pleural injury	0	0
Time spent	46 s	142 s

## Data Availability

All data derived from this study are presented in the text.
